# Response to letter to the editor regarding “Acute mountain sickness among tourists visiting the high-altitude city of Lhasa, Tibet, China at 3658 m above sea level: a cross-sectional study”

**DOI:** 10.1186/s13690-017-0188-6

**Published:** 2017-05-01

**Authors:** Gonggalanzi, Per Nafstad

**Affiliations:** 1 Tibet University Medical College, No.1 South Luobulinka Road, Lhasa, 850002 Tibet China; 2 Institute of Health and Society, University of Oslo, P.O. Box 1130 Blindern, Oslo, 0318 Norway; 3 Division of Epidemiology, Norwegian Institute of Public Health, Oslo, Norway

**Keywords:** Acute mountain sickness, Tourists

## Abstract

We kindly thank the journal for the opportunity to respond to the recent comments made regarding our manuscript entitled “Acute mountain sickness among tourists visiting the high-altitude city of Lhasa, Tibet, China at 3658 m above sea level: A cross-sectional study”.

We thank Gaurav Sikri and Srinivasa Bhattachar for their interest [1] in our recent work [2]. Their first comment is about the participants’ traveling history and ascent profile, which we agree is an important issue. As reported in the paper 47.3% arrived by air and 52.7% not by air. We do not have a detailed travel history for each individual. What we know is that it takes about 2 h by plane from lowland China to Tibet, and that it takes 2–4 days by train/bus/car. Those arriving by plane therefore definitely have a more steep accent profile than the others which we believe could explain why those arriving by plane had a higher Acute Mountain Sickness (AMS) prevalence than the other group.

The diagnosis AMS is based on non-specific symptoms which also could have other etiologies than altitude exposure. The questionnaire we used in our study is based on the globally used Lake Louise Scoring System (LLSS) which provides an AMS diagnostic standard [3, 4]. According to the literature, and since the 1991 Lake Louise consensus on AMS, most researchers use LLSS or LLSS based questionnaires. Therefore, we have strictly followed the Lake Louise Scoring System for defining AMS in this study. We acknowledge that there is a general challenge to decide if all the reported symptoms are strictly related to high altitude exposure and how different experiences during the journey could affect this. However, we are in doubt how to use travel history and especially the information on travel history available in our study to separate real AMS-related symptoms from symptoms of potential other origins if such cases exist.

We agree that valuable information could be lost when combining prophylactic drugs into one group. We showed the distribution of the different groups of drug users in the paper. The reason why we decided to treat users of prophylactic substances as one group was that we were not able to show any differences in prevalence between users of the different substances. Steroids and nifedipin were used by too few persons and we could not identify differences between users of acetazolamide and Chinese medicine either. As we have discussed in the paper, potential selection processes will make it challenging to draw conclusion about prophylactic effects based on a cross sectional descriptive survey like this. Gaurav Sikri and Srinivasa Bhattachar’s comment have also made us reflect if the term prophylactic drugs are the most correct expression to use in this setting. What we actually have information about is users of drugs taken for prophylactic purposes.

Most of the participants that developed AMS reported early onset symptoms as described in the paper. We did ask specifically about when symptoms started. The participants had alternative boxes to tick off including boxes for symptoms start less than 12 h and 12–24 h after arriving Lhasa. We did not give specific instructions about when to fill in the information only that the questionnaire should be returned within 3 days. Of course it is possible that some participants were not able to recall precisely when the symptoms started but we were and are of the opinion that within a few days one will remember quite accurately when one starts to get symptoms.

## Abbreviations

AMS: Acute Mountain Sickness
LLSS: Lake Louise Scoring System
